# Obstacle Detection Using a Facet-Based Representation from 3-D LiDAR Measurements

**DOI:** 10.3390/s21206861

**Published:** 2021-10-15

**Authors:** Marius Dulău, Florin Oniga

**Affiliations:** Computer Science Department, Faculty of Automation and Computer Science, Technical University of Cluj-Napoca, 400114 Cluj-Napoca, Romania; mariusdulau9@gmail.com

**Keywords:** LiDAR point cloud, obstacle detection, object contour, facet representation

## Abstract

In this paper, we propose an obstacle detection approach that uses a facet-based obstacle representation. The approach has three main steps: ground point detection, clustering of obstacle points, and facet extraction. Measurements from a 64-layer LiDAR are used as input. First, ground points are detected and eliminated in order to select obstacle points and create object instances. To determine the objects, obstacle points are grouped using a channel-based clustering approach. For each object instance, its contour is extracted and, using an RANSAC-based approach, the obstacle facets are selected. For each processing stage, optimizations are proposed in order to obtain a better runtime. For the evaluation, we compare our proposed approach with an existing approach, using the KITTI benchmark dataset. The proposed approach has similar or better results for some obstacle categories but a lower computational complexity.

## 1. Introduction

Autonomous vehicles use sensors for environment perception in order to detect traffic participants (pedestrians, cyclists, vehicles) and other entities (road, curbs, poles, buildings). A perception system can consist of a standalone sensor or a combination of sensors, mainly camera, radar, and LiDAR. LiDAR sensors are used for perception, mapping, and location. For the perception part, the algorithms that process the data from this type of sensor focus on object detection, classification, tracking, and prediction of motion intention [1].

Generally, the algorithms used for object detection extract the candidate objects from the 3-D point cloud and determine their position and shape. In a 3-D point cloud obtained with a LiDAR sensor for autonomous vehicles, objects rise perpendicularly to the road surface, so the points are classified as road or non-road points. After separating the non-road points from the road ones, objects are determined using grouping/clustering algorithms [1]. Usually, objects detected in the scene are represented with a rectangular parallelepiped or cuboid.

Facet detection is a particular variant of object detection. The facet-based representation describes objects more accurately. With the cuboid representation, an object has a 3-D position, size, and an orientation. With facets, the object is decomposed into several component parts, each part having its own position, size, and orientation. When the vertical size of the facets is ignored, the representation is the standard polyline (a chain of line segments describes the object boundaries in the top/bird eye view).

**Figure 1:**
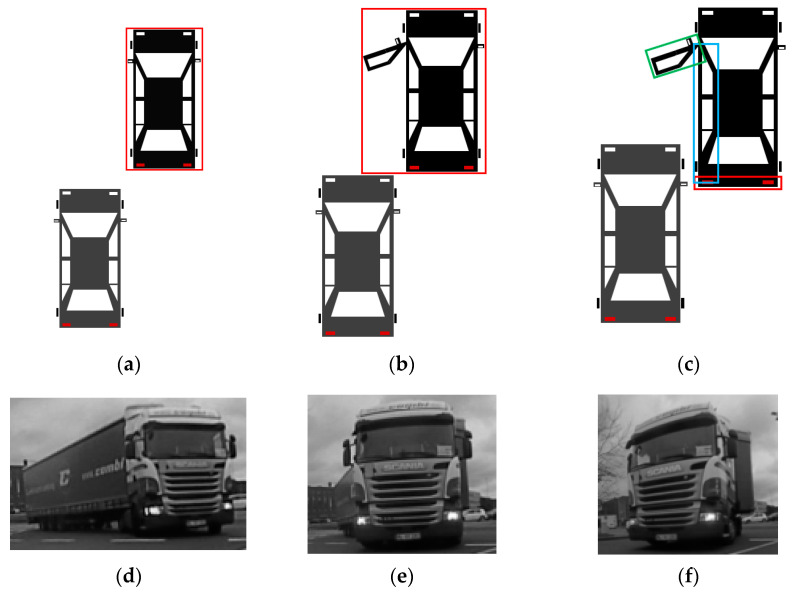
Potential application: (**a**–**c**): Facet-based representation used in automatic emergency braking situations (top view visualization). (**a**): The gray car detects the black car (red bounding box). (**b**): The gray car brakes to the rear of the black vehicle with the door open (if the car is detected as the red bounding box). (**c**): The gray car can perform a smoother braking if the black car is represented by its visible facets (each facet with a different color). (**d**–**f**): A large articulated vehicle cannot be modelled as an oriented cuboid during cornering maneuvers.

For obstacles that have a cuboidal shape, the volume occupied can be accurately represented with an oriented cuboid. However, for other non-cuboidal shapes, facets provide a better representation for the occupied areas, visible from the perspective of the ego car. The facet/polygonal representation provides a better localization for the boundaries of non-cuboidal shaped obstacles. This allows a more accurate environment representation, thus improving potential driving assistance functions. For example, for the automatic emergency braking functionality, there might be a situation where a car is parked and another car comes from behind, overpassing the parked one. In the parked car, the driver’s door is opened suddenly. With the cuboid representation of the stationary car, the moving car will brake immediately to avoid the collision (because the actual free space is inside the cuboid representation of the parked car). With the facet-based representation, the rear vehicle will perform a less sudden braking and can even avoid the collision through automated steering (Figure 1a–c).

Another justification for facets is the more accurate representation of infrastructure objects that have a curved shape, such as particularly shaped fences or buildings (see Section 4). Additionally, large articulated vehicles like busses, trams, or heavy goods vehicles cannot be modelled by a single oriented cuboid during cornering maneuvers (Figure 1d–f). Some vehicles are designed with strongly curved frontal/rear profiles. For these models, the cuboid representation might overestimate the occupied space at the vehicle’s extremities (an example is shown in Section 4). At the same time, this representation is more suitable for obstacle classification, as it captures more accurate information about the obstacle shape.

In this paper, we propose a new processing pipeline for detecting obstacles with a facet-based representation. The main steps of the approach proposed in this paper are ground points detection and removal, grouping of the remaining points into objects (or clusters), and determining the facet representation for each obstacle in the scene. For the ground detection step, we use an existing method, with an improvement to increase the processing speed while preserving the quality of the results. The main contributions are for the clustering and the facet detection steps. For clustering, we propose a new method based on intra- and inter-channel clustering. For the facet detection step, we propose a method based on RANSAC that iteratively extracts the facets from the contour of each object. We use the datasets available from the KITTI benchmark for testing the proposed approach.

The paper is organized as follows: Section 2 shows the related work for ground/road detection, clustering, and facet detection. In Section 3, we underline the contributions and describe the proposed approach. In Section 4, we present the results and perform an evaluation of the proposed approach. Section 5 concludes the paper.

## 2. Related Work

### 2.1. Ground Point Detection

Based on the specific approach, methods for ground point detection can use mainly geometric-based reasoning or neural networks for processing. Relevant geometric-based methods for road/ground detection were presented in [2,3,4]. Neural network-based methods for road detection were presented in [5,6,7,8,9,10,11]. Most of these methods typically represent the 3-D point cloud as images, which are provided as input to the neural networks that detect the drivable area. Evaluation is commonly done on the KITTI road dataset.

A traditional method to detect the ground points was presented in [3], where Chu et al. used the angle of the slope computed with two consecutive points along the same channel (azimuth value). All the points from a channel are scanned from near to far, and slopes are computed. Based on the slope, each point is labeled as ground point or obstacle. Another way to detect the road points was presented in [4], where Asvadi proposed an algorithm that determines the surface of the road in four stages: slicing, gating, RANSAC plane fitting, and validation.

In [9], Velas et al. converted the 3-D points into a dense multi-channel image and used a convolutional neural network (CNN) to detect the ground points. The evaluation was performed on ground truth from KITTI. A similar approach was presented in [10], where the authors have more channels for the dense image and the CNN runs on field-programmable gate array (FPGA). In [11], the road points are segmented using a network architecture different from the ones presented in [9,10]. The LiDAR data is organized as images representing the top-view of the scene and the information encapsulated is limited to 40 m in front and 10 m on each lateral side of the car.

### 2.2. Object Detection

For object detection, in [12], Klasing et al. use a kd-tree for storing the third indoor point cloud and cluster the points using the radially bounded nearest neighbor (RBNN). For defining objects, voxels are used in [4,13]. In [14], Bogoslavskyi and Stachniss use the range image corresponding to the scene and a breadth-first search (BFS) algorithm to create the object clusters. In [15], the information about the color is used to build the clusters.

The authors of [16] propose an object detection approach using a CNN with three layers called LaserNet. The image representation corresponding to the environment is created using the layer identifier and the azimuth angle. For each valid pixel, the distance to the sensor, height, azimuth, and intensity are saved, resulting in a five-channel image, which is the input to the CNN. The network provides several cuboids in the image space for objects and, to solve this, mean-shift clustering is applied to obtain a single cuboid. In [17], an improvement is proposed for the CNN from [16] in order to process information about the pixels’ color, so, in addition to LiDAR, a color camera is also used.

In [18], SqueezeSeg, a network for object detection, is proposed. The point cloud from LiDAR is projected onto a spherical representation (360° range image). The network creates label maps, which tend to have blurry boundaries produced by the loss of low-level details in the max-pooling operations. In this case, a conditional random field (CRF) is used to correct the result of the CNN. The paper presents results for cars, pedestrians, and cyclist from the KITTI dataset. In [19], another network (PointPillars) provides results for cars, cyclists, and pedestrian detection. The point clouds are converted into images in order to use the neural network. The neural network has a backbone to process 2-D images and a detection head based on a single shot detector (SSD), which detects the 3-D bounding boxes.

The authors of [20] propose a real-time framework for object detection that combines camera and LiDAR sensors. The point cloud from LiDAR is converted into a dense depth map, which is aligned to the camera image. A YOLOv3 network is used to detect objects in both camera and LiDAR images. An Intersection-over-Union (IoU) metric is used for fusing the bounding boxes of objects from both sensors’ data. If the score is below a threshold, then two distinct objects are defined; otherwise, one single object is defined. Additionally, for merging, a Dempster–Shafer evidence was proposed. The results were evaluated on the KITTI dataset and Waymo Open dataset. The detection accuracy was improved by 2.84% and the processing time of the framework was 0.057 s.

The authors of [21] present a method for the detection of far objects from dense point clouds. In the far range, in a LiDAR point cloud, objects have few points. The Fourier descriptor is used to describe a scan layer for classification and a CNN is used. First, in the pipeline, the ground is detected. Then, objects are extracted using Euclidean clustering and separated into planar curves (for each layer). The planar curves are matched in consecutive frames, for tracking.

In [22], the authors propose a network for object and pose detection. The network consists of two parts: a VGG-based object classifier and a LiDAR-based region proposal network, the last one identifying the object position. Like [18,19], this method performs car, cyclist, and pedestrian detection. The proposed method has four modules: LIDAR feature map complementation, LIDAR shape set generation, proposal generation, and 3-D pose restoration.

Other methods based on neural networks, for 3-D object detection, were presented in [23,24,25,26,27,28]. In these approaches, single-stage or more complex (two-stage pyramidal, in [24]) networks are proposed and evaluated on the KITTI dataset. In [25], the point cloud is converted into a range image and objects are detected based on the depth feature. Camera data is fused with LiDAR data in order to detect better objects [26]. In some works, the detection of objects is approached by performing semantic segmentation on LiDAR data [29,30] or camera-LiDAR fused data [31].

In [32,33], the authors underline that the cuboid representation is not suitable for objects because it overestimates the space occupied by non-L-shaped objects, such as a circular fence or a more complex building. A better representation of the objects is by polylines or facets.

### 2.3. Facet Detection

The authors of [34] present facet detection for urban buildings from LiDAR point clouds. Their approach uses range images in order to process all the points of an object faster. The depth image is filtered to remove noise, after which it is binarized in order to apply morphological operations to fill the gaps in objects. The next step is to apply a Laplace filter to determine the contour of the object. After obtaining the contour, the vertical lines separating adjacent facets of the buildings are determined using defined formulas.

A different method to detect facets was presented in [35], where the RANSAC method is used for fitting a plane to each object side. All points are used in the processing step. The problem of the intersection of the planes is approached in order to correctly assign a point to a facet. For intersecting facets, the surface residuals are calculated using the point of intersection and the points immediately adjacent. The standard deviation values for both sets of residuals are then calculated and the intersection point is assigned to the facet that has the lowest value of the standard deviation.

In [33], objects are represented as polylines, a polyline segment being the base structure of a facet. Their quantitative evaluation is based on the orientation angle of the object and the results show that representation using polyline is closer to the ground truth than the cuboid representation. A complex representation based on polygons is proposed in [36], by modelling the 3-D points cloud as a polygonal (triangular) mesh, with potential applications for aerial depth images, traffic scenes, and indoor environments.

## 3. Proposed Approach for Obstacle Facet Detection

**Figure 2:**
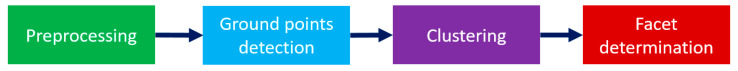
System architecture.

The proposed system (Figure 2) consists of four steps: LiDAR data preprocessing, ground point detection, creation of object instances via clustering, and facet detection for each object.

For the preprocessing step, the 3-D point cloud is enriched with the layer and channel identifiers, and the relevant coordinates are selected for each 3-D point, which will allow faster processing in the next steps. For the ground detection step, the method from [3] is selected, but it is improved to increase the processing speed while preserving the quality of the results. For clustering, we propose a new method based on intra- and inter-channel clustering, which in comparison with an existing octree-based approach, is faster and requires less memory. For the facet detection step, we propose a new method, which uses RANSAC as in [35], but has as input the contour of the object, not all the visible object points.

### 3.1. Preprocessing

The information about the channel and layer identifier is not present in the KITTI dataset, so we determine it. A 360° LiDAR provides a circular point cloud, and we divide it into more channels. We calculate a channel ratio depending on the number of channels we want. The number of channels is a multiple of 360:

(1) $$\mathrm{channel}\;\mathrm{ratio}=\frac{360}{\mathrm{channels}}$$

For a 3-D LiDAR point $A\left(x_{A},y_{A}\right)$, we calculate the azimuth angle. If the angle value is negative, then we add 360:

(2) $${\mathrm{azimuth}}_{A}=\tan^{-1}\left(y_{A},{\;x}_{A}\right)$$

The channel identifier is determined from the ratio of the azimuth angle and the channel ratio (or horizontal angular resolution):

(3) $$\mathrm{channel}\;\mathrm{ID}=\frac{\mathrm{azimuth}}{\mathrm{channel}\;\mathrm{ratio}}$$

In the KITTI dataset, the points are organized from the top layer to the bottom layer, in ascending order of the vertical angle, from 0° to 360°. The layer identifier is incremented each time a change is detected from a negative to a positive value of the azimuth angle (before adding 360 for the negative values).

**Figure 3:**
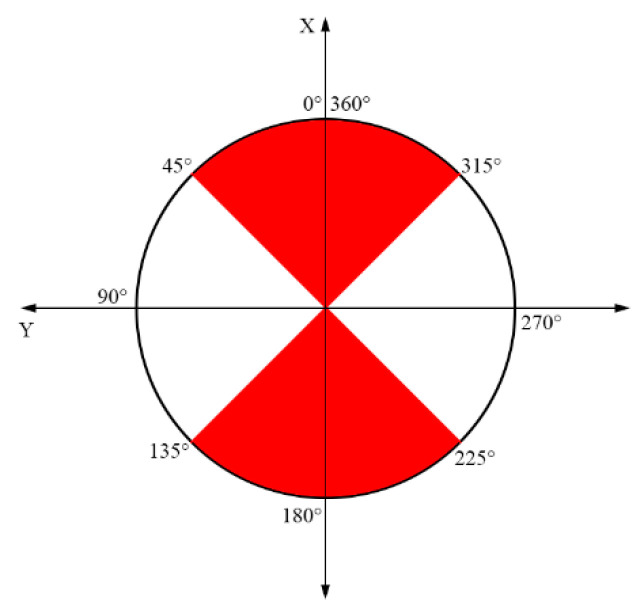
Channel values representation in a point cloud (**top** view). The red area is where the value for X is bigger than the value for Y for the same point; the white area is where the value for X is smaller than the value for Y.

The reference system for LiDAR measurements has the following meaning for the axes: x—longitudinal/depth, y—lateral (towards the left), z—vertical. For each point, two coordinates are relevant for the next processing steps: the elevation and the distance to the sensor in the horizontal plane. The elevation is the vertical location of a point, given by the Z coordinate, and the distance or horizontal location depends on the region where the point is registered. Depending on the 3-D point channel orientation, the point can be considered predominantly in the front/rear of the sensor or on the left/right sides (Figure 3). For points placed in the front/rear, the X coordinate (larger than Y) is selected as the horizontal distance, while for the other points, the Y value is considered as the horizontal distance. These values will be used in the ground segmentation and in the clustering steps, in order to have a low computational complexity.

### 3.2. Ground Point Detection

**Figure 4:**
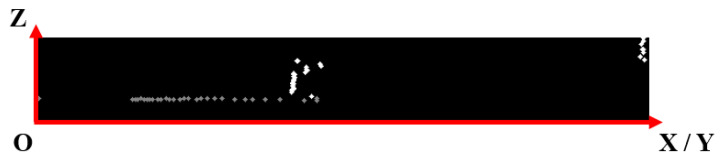
Side view of a channel. Detected ground points are colored with gray. X/Y means either the *x*-axis or the *y*-axis value is used, depending on the specific channel orientation.

**Figure 5:**
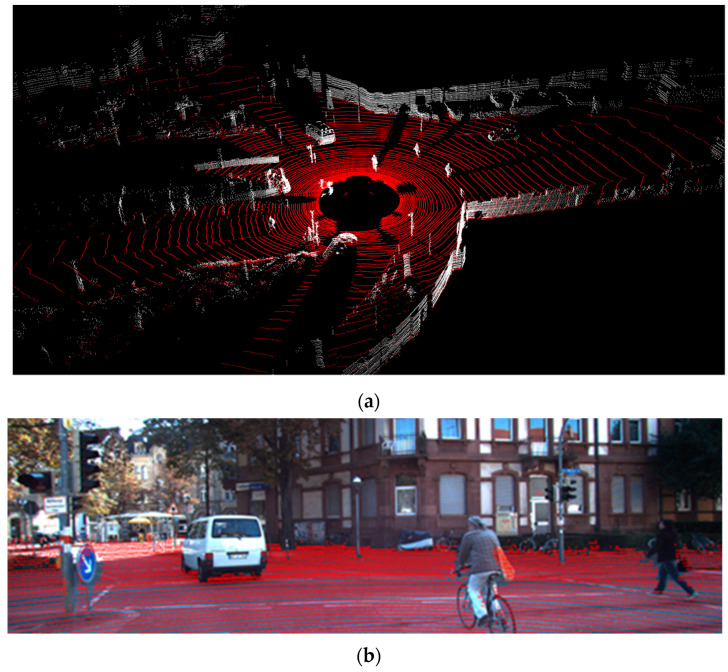
Ground detection. (**a**): Results shown in 3-D view. (**b**): Results overlay over the corresponding camera image.

For the ground point detection step, we used the algorithm presented in [3], where points are analyzed along each channel. The vertical angle between 2 consecutive points is used for discriminating between road and obstacle points. If the angle is below a threshold, then the point is classified as ground. In [3], the angle is calculated using the $\sin^{-1}$ formula, which implies a division to a square root, as in Equation (4). Instead of $\sin^{-1}$, we use $\tan^{-1}$ to get rid of the division with the square root and to obtain less computations (processing times in Section 4). Equations (4) and (5) present the formulas used to compute the angle. In our implementation, we used the row (elevation) and column (horizontal distance) values specified in Section 3.1. A representation of a channel from the side view (with ground points detected) is shown in Figure 4. Figure 5 presents the final ground detection result:

(4) $$\alpha =\sin^{-1}\left(\frac{p_{2}.row-p_{1}.row}{\sqrt{{\left(p_{2}.col-p_{1}.col\right)}^{2}+{\left(p_{2}.row-p_{1}.row\right)}^{2}}}\right)$$

(5) $$\alpha =\tan^{-1}\left(\frac{p_{2}.row-p_{1}.row}{p_{2}.col-p_{1}.col}\right)$$

### 3.3. Clustering

After ground detection and removal, the remaining obstacle points must be grouped into clusters. As a baseline, we started with an implementation with octree point cloud structuring and clustering based on the RBNN algorithm [12]. A stable octree implementation is defined on the maximum volume of interest. Each octree node generates additional data to the point cloud: the node root, the node dimensions, and the point storage. The smaller a node is, the more nodes an octree has, and the more memory is used to represent the point cloud. For the octree approach, a volume is defined that encapsulates the entire point cloud. The total length and width are set to 180 m and the height is set to 4.05 m. The octree leaf has a size of 0.15 m for each side. By dividing the volume of interest to the octree leaf, we obtain a number of 1200 × 1200 × 27 = 38.8 million octree leaves in the structure. Each octree leaf node has information about its center, size, and the points it contains. So, even if only the octree leaves are considered, they require a lot of memory. Another disadvantage is the placing of a point in an octant, which requires computations to select the right octant. As an advantage, the octree structure is suitable for parallelization and is used to obtain better runtimes.

In order to reduce the computational complexity and the memory requirements, we propose a new approach for clustering, which handles data in 2-D (in each channel space), not in 3-D as in the case of the octree. The method is described in the next paragraphs and as pseudo-code in Algorithm 1.
**Algorithm 1.** Clustering based on intra- and inter-channel processing.1:**for each** channel *c*2:  create clusters of close points3:  **for** all pairs (g_i_, g_j_) of clusters4:    **if** intersect(g_i_, g_j_) or closeClusters(g_i_, g_j_) **then**5:      merge(g_i_, g_j_)6:
  **end if**
7: end for8: **for each** cluster g_i_
∈ c9:  **for** k = 1,PREVIOUS_CHANNELS_TO_CHECK10:   **for each** cluster g_j_
∈ c_c.index-k_11:    **if** doClustersOverlap(g_i_, g_j_) or closeClusters(g_i_, g_j_)12:      sc = supercluster(g_j_.superClusterIndex)13:      sc.update(g_i_)14:
    **end if**
15:   end for each16:  end for17:  **if** no matching cluster **then**18:   createNewSuperCluster()19:
  **end if**
20: **end for each**21:**end for each**

**Figure 6:**
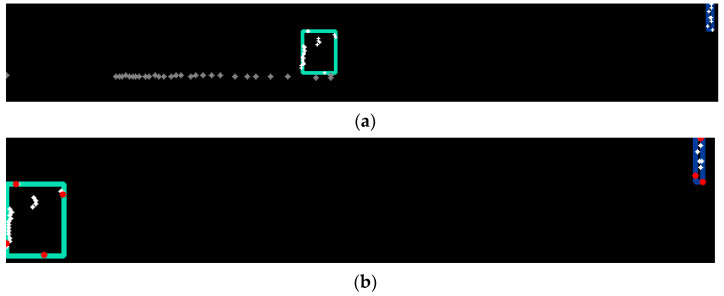
(**a**): Object primitive clusters in a channel. (**b**): Boundary points of a cluster shown in red.

First, intra-channel analysis is performed to compute channel-level primitives of obstacles. In each channel, we determine the clusters of points (Figure 6a). For each cluster, the extreme points on each axis are selected (Figure 6b). These points provide the bounding boxes of the intra-channel clusters and are used further in object instance creation. After the initial channel clusters are determined, we look for close or overlapping clusters in the same channel and merge them. We consider that two clusters are close if the distance between them is smaller than the DISTANCE_BETWEEN_CLUSTERS value (=0.15 m).

**Figure 7:**
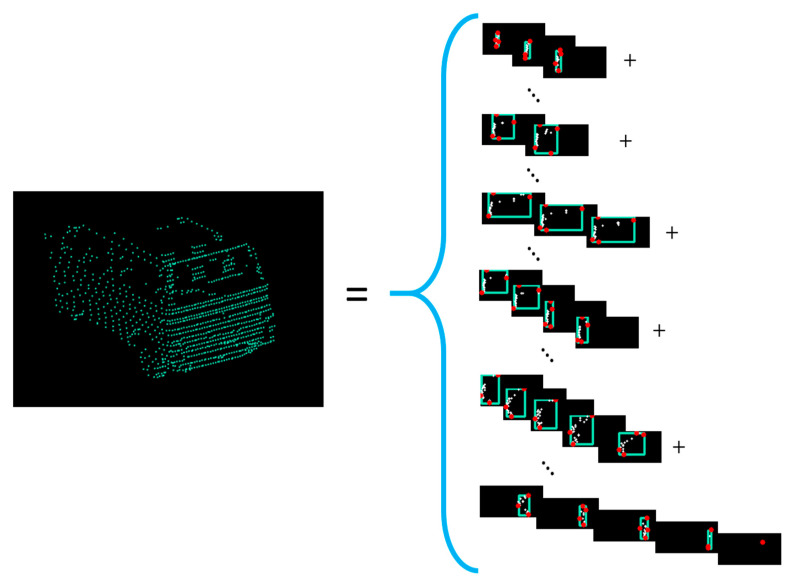
The creation of the intra-channel clusters for a van is presented. Each cluster is represented by a rectangle (delimited by the most extreme points—red dots) in its channel side view. The clusters have different sizes and are intersecting or are close to the previous channel clusters.

Next, inter-channel clustering is performed. For the current cluster, we check in the previous PREVIOUS_CHANNELS_TO_CHECK (=7) channels for close clusters or for clusters that intersect each other, in order to grow the object, also called a supercluster (Figure 7). There are situations where a cluster can be assigned to two objects previously defined. In this case, we merge the objects detected in the previous channels. For instance, this situation can happen when there are points from inside of the objects (measurements inside of vehicles, through the windshield) and the next channel cluster is from the outside of the object.

**Figure 8:**
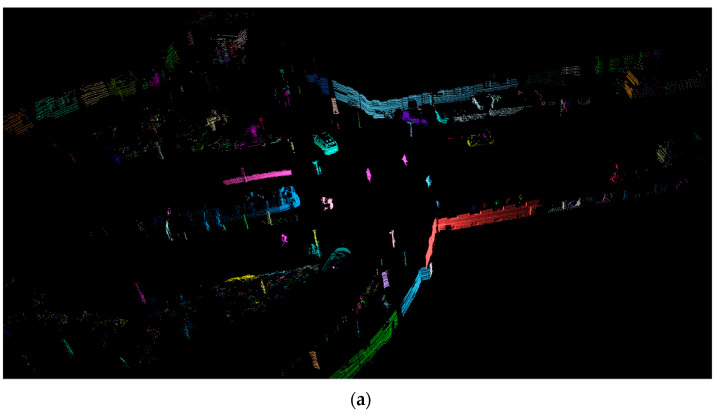

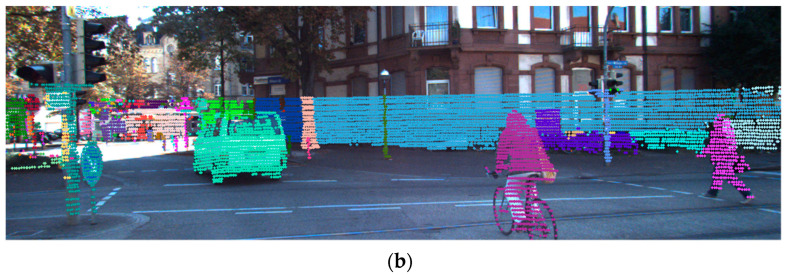
Clustering results. (**a**): Object clusters in point cloud, with distinct color per cluster. (**b**): Detected clusters projected on the corresponding camera image.

The results of the proposed clustering method are shown in Figure 8. The advantage of this channel-based method compared to the octree one is less memory utilization, as no global representation (the octree) of the measurements is required.

### 3.4. Facet Determination

**Figure 9:**
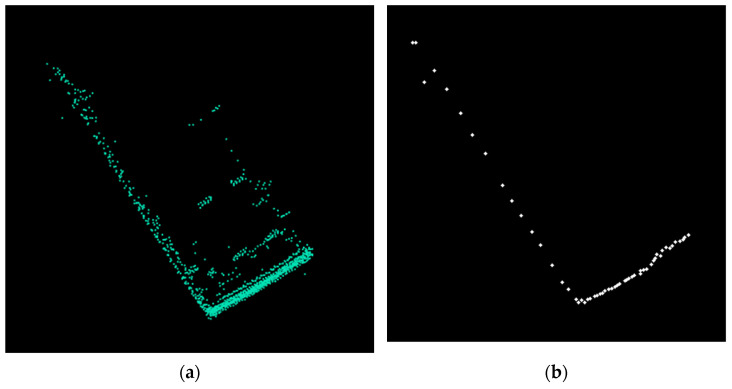
(**a**): Top view of a van. (**b**): Contour obtained from clustering.

After obtaining the objects instances from the clustering stage, facet detection is performed. In the clustering stage, each object is created by grouping multiple primitive clusters from adjacent channels. Each primitive cluster has delimiter points relative to the ego car position. These points are the closest ones to the sensor position. By selecting these delimiter points, the contour (Figure 9) of each object from the scene is extracted.

The algorithm (Algorithm 2) for facet detection is based on the following steps: contour point filtering, the base line of the facet construction, and new facet creation or merging with the previous facet if the current and the previous facet have similar orientations. For the first step, as the points from LiDAR are noisy, we apply a triangular filter to correct the position of each point. The filter has as input 5 consecutive points (two previous, one current and the next two, Equation (6)) and the one from the middle will be corrected:

(6) $$x_{\mathrm{new}}=\frac{x_{t-2}+2x_{t-1}+3x_{t}+2x_{t+1}+x_{t+2}}{1+2+3+2+1}$$

**Algorithm 2.** Facet detection.1:**for each** object cluster obj2: filterNoisePoints(obj.contour)3: **while** obj.contour ≠ empty4:  facetPoints = {obj.contour.p1, obj.contour.p2}5:  facetLine = calculateLineCoefficients(facetPoints)6:  facetLine.outlierPercent = 1007:  **while** outlierPoints ≤ MAX_CONSECUTIVE_OUTLIERS8:   d = distance(currentPoint, facetLine)9:   **if** d ≤ MAX_DISTANCE_TO_FACET **then**10:    facetPoints = facetPoints ∪ currentPoint11:**   else**12:    facetLineAux = calculateLineCoefficients(facetPoints)13:   **if** angleChanged(facetPolyLine) **then**14:    createFacetAndAddToObject(facetPoints, facetLine)15:
   **end if**
16:   **if** facetLineAux.outlierPercent < facetLine.outlierPercent or facetLineAux.outlierPercent < outlierPercentThreshold **then**17:    facetLine = facetLineAux18:    **if** distanceFromPointToLine (currentPoint, facetLineAux) ≤ MAX_DISTANCE_TO_FACET **then**19:     facetPoints = facetPoints ∪ currentPoint20:     resetOutlierPoints()21:
    **end if**
22:
   **else**
23:    incrementOutlierPoints()24:
   **end if**
25:  end while 26:  createFacetAndAddToObject(facetPoints, facetLine)27:  **if** angleBetweenLast2Facets(facets) ≤ ANGLE_DIFF **then**28:   mergeLast2Facets(facets)29:
  **end if**
30:  **end while**31:**end for**

A facet is based on a line segment. For determining a facet, we use the random sample consensus (RANSAC) algorithm to determine the line with its coefficients. The RANSAC algorithm has a fixed upper limit for the number of iterations in order to improve the runtime. Taking into account the specifics of the measurements (percentage of outliers), the number of iterations is up to 20. First, we determine the coefficients of the line with the first 2 points from the object contour. Then, we test if the next points fit the line in order to know when to stop and to create a new facet. The fitting criteria is done by comparing the distance from the point to the line with a predefined threshold MAX_DISTANCE_TO_FACET (=0.08 m). If it fits, then the point is added to the facet; otherwise, the coefficients of the line are recalculated with the existing valid points. After the new coefficients are calculated, we check if the new line has more inliers. If it has, then we keep the new coefficients; otherwise, the previous ones are kept, and the number of consecutive outliers is increased. If the coefficients for the base line of the facet are updated, then we check if the current point now fits the line. If it does not fit, we increment the number of outliers; otherwise, we reset the number of consecutive outliers to 0.

A new facet is created when we have a number of consecutive outliers above the predefined threshold MAX_CONSECUTIVE_OUTLIERS (=4). Another case in which a new facet is created is when the recalculated coefficients show a big variation in the orientation angle. This situation appears around objects’ corners. The angle difference is compared to a threshold, ANGLE_DIFF (=10°).

Before adding the created facet in the facet set of the object, we perform an angle check with the last facet from the set. If the angle is below the threshold, then we merge and update the last facet with the current one (the points of the current facet are added, and the base line of the facet is recomputed).

After the steps described above, we resume the process of new facet creation for the next points until all the current contour points are scanned.

The facet detection can be further optimized for speed, by further sampling the contour points. In our experiments, we used a sampling rate of 2. In this way, less computations are performed, and the shape of the object does not change much. This step is inserted after noise filtering stage.

Finally, each facet is assigned a height equal to the object’s height.

## 4. Evaluation and Results

The implementation was done in C++, and OpenMP was used to parallelize the code on multiple cores. The system used for testing is equipped with an Intel Core i5-8300H CPU and 8GB RAM. The runtime for each part of the system was measured on sequential execution and also on parallel execution. For parallel execution, we used four threads, with appropriate implementations. All the runtimes are expressed for the entire 360° point cloud’s processing. All the runtimes presented are based on 252 scenes from [9]. For each scene, we performed 10 measurements and calculated the mean. The final average runtime of each processing step was calculated using the mean runtime from each scene.

### 4.1. System Parameters

The parameters used in our implementation are listed in Table 1. For ground detection, the parameters are the same as those from [3].

The number of channels parameter determines the angle aperture of the point cloud sector (depends on the LIDAR angular resolution). With 1800 channels, a sector has an angle of 0.2°. If the angle is bigger, then more points will be embedded in the same channel and the ground detection algorithm will not work as precisely because more points can be on the same layer (aliasing). If the angle is smaller, then a channel will have fewer points making the ground detection algorithm more precise, as a single point from each layer will be selected.

The parameter DISTANCE_BETWEEN_CLUSTERS influences the minimum distance between the final objects: the bigger the value is, the more objects will be combined in one single object. The next parameter, PREVIOUS_CHANNELS_TO_CHECK, is used to check for intra-clusters in occluded objects. The higher the value is, the more previous consecutive channels will be checked, but there is a risk of combining two objects into one (e.g., two parallel cars).

The distance between a point and the support line of the facet is represented via the MAX_DISTANCE_TO_FACET parameter. This parameter determines if a point is an inlier or an outlier. A higher value will allow more inliers, but the base line of the facet determined by RANSAC might create wider facets. The angle between the facets is used to check if they can be fused, as the first points from the new facet are outliers for the previous one. If ANGLE_DIFF has a lower value, the fused facet still represents a correctly occupied volume. Otherwise, the facet will overestimate the volume occupied by an object part. Maximum RANSAC iterations specify how many trials should be made to find the best coefficients of the line. The higher the value, the more iterations are performed. This means a longer execution time, but the results are more accurate.

### 4.2. Ground Point Detection

**Figure 10:**
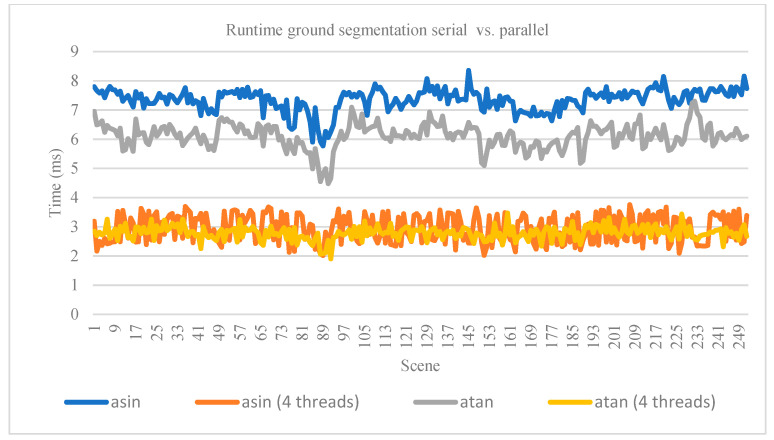
Runtime comparison graph for ground detection methods on 252 scenes.

For ground detection, we used the annotated files from [9] consisting of 252 scenes. We associates the files with the scene from the KITTI tracking dataset [37]. The quality of ground detection was measured using accuracy, precision, recall, and f1-score metrics. We observed that the improvement with $\tan^{-1}$ has a better runtime and the quality of detection is not decreased. Our results are shown in Table 2 and Table 3—quantitative evaluation, and Table 4 and Figure 10—runtime. In Table 2, the true positive represents the points (all the points from the 252 scenes) that are correctly classified as ground, and true negative represents the points that are classified correctly as obstacle. False positive values represent points classified as ground but are actually a type of obstacle. False negative points are the points classified by the algorithm as an obstacle but are actually a type of ground.

### 4.3. Clustering

**Figure 11:**
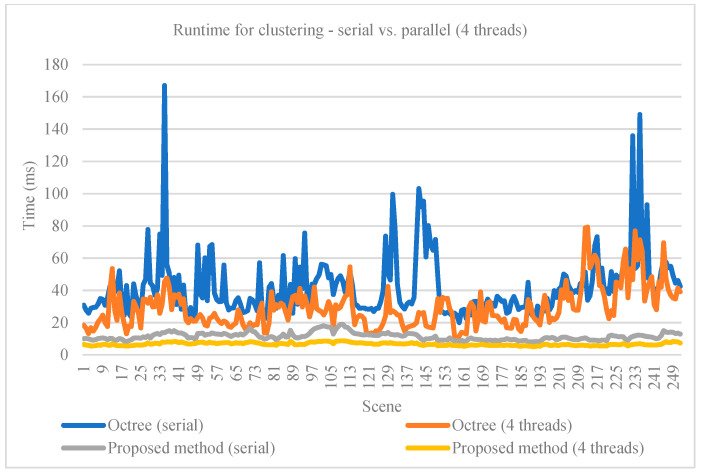
Runtime comparison graph for clustering methods on 252 scenes.

For the clustering method, we compared the runtime of the proposed implementation with a method based on octree structuring [13] and RBNN used for clustering [12]. Both methods’ runtime were evaluated on serial and parallel execution. The runtime is considered for the entire point cloud. Our method uses less memory and is faster, as it performs fewer load and store operations in contrast with the octree representation. The runtimes are shown in Table 5 and Figure 11. Quantitative comparison at this stage between these clustering methods is not possible, as they output clusters (sets of points belonging to the same obstacle) without a corresponding oriented cuboid (the ground truth available in the KITTI set).

**Figure 12:**
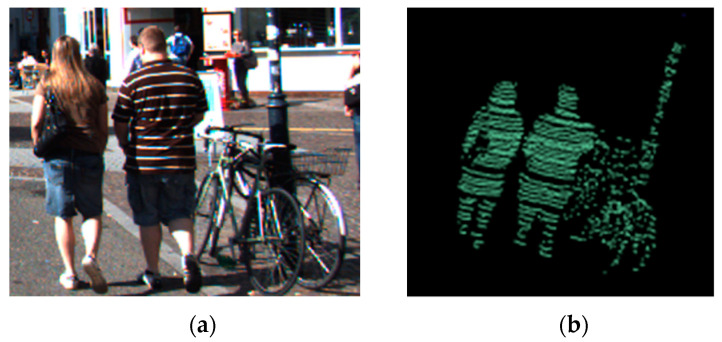
Close multiple objects clustered as one single object. (**a**): Image with close multiple objects. (**b**): Single cluster created—point cloud view (same label for all the points).

As our method for clustering is mainly based on adjacency criteria, multiple close objects might be clustered into one single object (see an example in Figure 12).

### 4.4. Facet Detection

In order to evaluate our method for facet detection, we implemented the method from [34] and adapted it to all types of objects. In [34], the method was proposed for extracting the facets of buildings from LiDAR range images and the parameters are suitable for that use case. We set new values for those parameters in order to work on all types of objects in the KITTI dataset. For example, in [34], a sliding window for scanning the range image was calculated as the ratio between the building width and grid size of the point cloud projection. In the KITTI dataset, there are objects of various sizes, smaller than buildings, so we set the size of the sliding window to five pixels.

**Figure 13:**
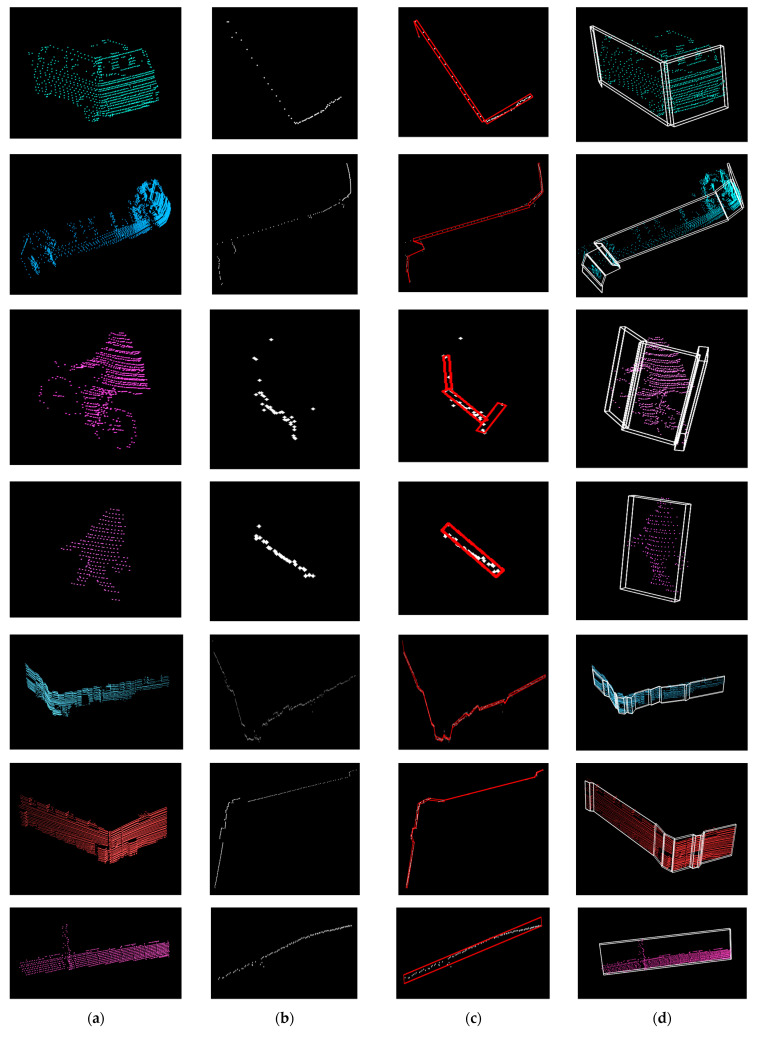
Facet detection on a van, tram, cyclist, pedestrian, buildings, and a wall. (**a**): Objects from the point cloud. (**b**): Contour of object (**top** view). (**c**): Facets (with red) over contour. (**d**): 3-D facets over objects.

**Figure 14:**
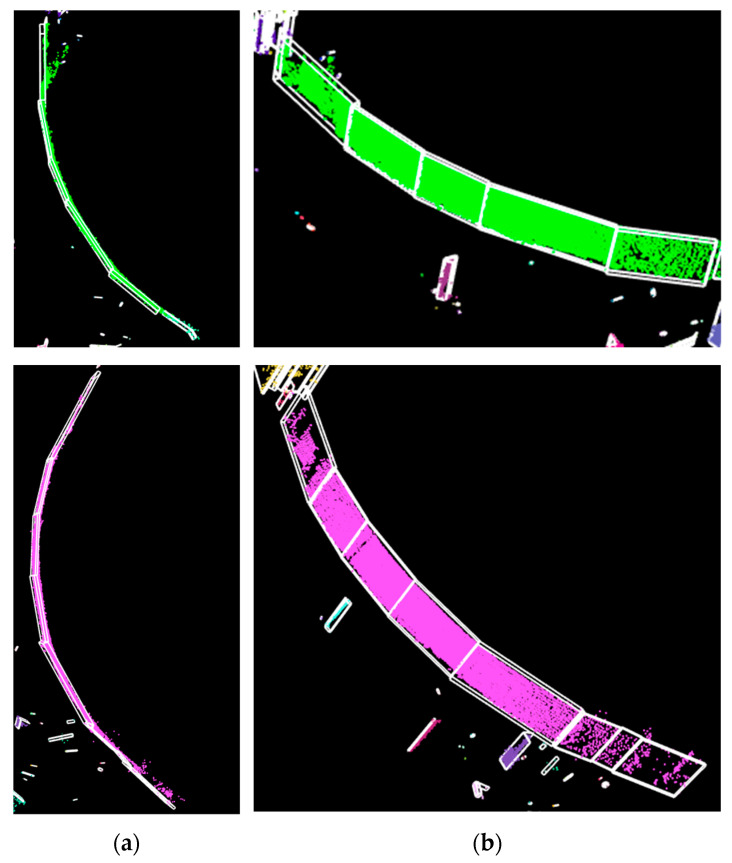
Circular fences. (**a**): **Top** view. (**b**): Perspective view. The detected facets are displayed (white).

The evaluation for facets was done on the KITTI object detection dataset consisting of 7481 scenes. The dataset has the following labels: car, cyclist, misc, pedestrian, person sitting, tram, truck, and van. Sample results are presented in Figure 13. Additionally, our method performs well for curved objects, particularly shaped fences (see Figure 14).

**Figure 15:**
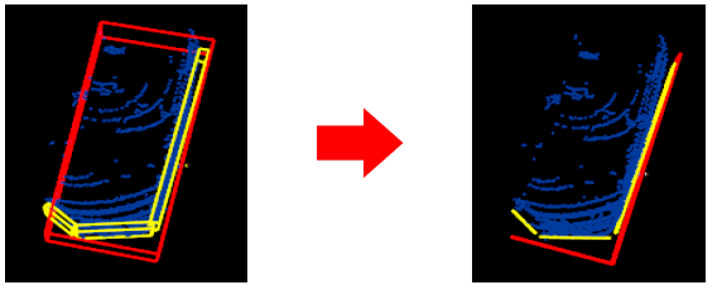
Facets extracted from the KITTI bounding box (red) and 3-D output facets (yellow) from our implementation are paired for comparison.

For the quantitative evaluation of our facet detection implementation, we use the 3-D bounding boxes from KITTI. From them, we extract the facets visible to the sensor (one or two facets), depending on the shape of the obstacle. Each detected facet is assigned to an extracted cuboid facet from KITTI (Figure 15).

**Figure 16:**
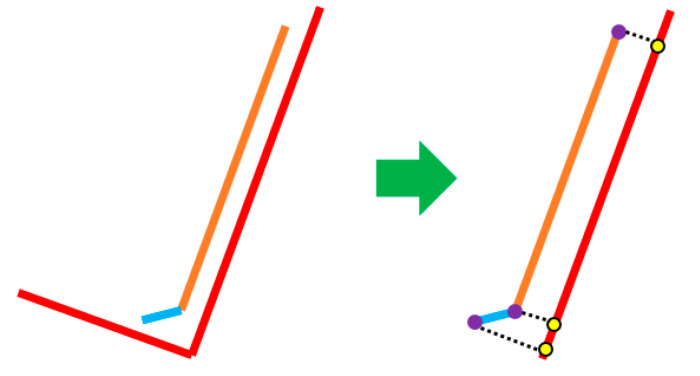
Projection of facets (orange and blue) on the KITTI-extracted face (with red).

A facet can be assigned to two KITTI cuboid facets. The next step is to project the detected facet to the assigned one (Figure 16) and calculate an IoU score. Considering the score of each facet from the object, we calculate an average IoU score per object. A facet detected by our algorithm can have a different orientation from the corresponding KITTI facet. To compensate for this, we apply a penalty when computing the IoU score. In Equation (7), obj is the object, F is the number of facets from our algorithm, K is the number of extracted facets from the KITTI bounding box, inter is the intersection function, and θ is the angle between our facet and the corresponding extracted facet from the KITTI cuboid:

(7) $${\mathrm{obj}}_{\mathrm{IoU}}=\frac{\sum_{i=1}^{F}\mathrm{area}\left(\mathrm{inter}\left(\mathrm{obj}.{\mathrm{facet}}_{i}\right)\right)*\cos\theta }{\sum_{j=1}^{K}\mathrm{area}\left(\mathrm{obj}.{\mathrm{kittiFacet}}_{j}\right)}$$

We compared the results of facet detection IoU from the adapted algorithm [20] with our proposed approach on the following classes: car, cyclist, misc, pedestrian, truck, and van. For the person sitting class, the clustering algorithm takes other objects into account, e.g., a table, and evaluation is not correct. We observed that the results for our method are similar or improved in comparison with the adapted method from [34] (Table 6). Additionally, the tram class was not considered, as the ground truth available was often split into several parts due to the large length.

**Figure 17:**
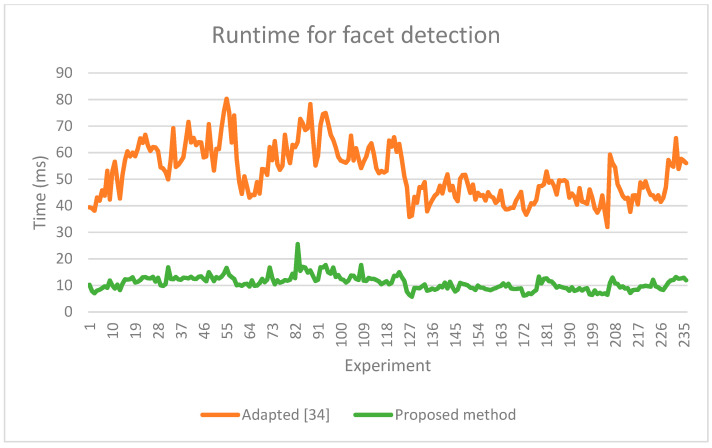
Runtime comparison graph for facet detection methods on 252 scenes.

As our method is faster (Figure 17, Table 7), we evaluated the runtime for the proposed facet detection step with a parallel execution implementation having four threads (Table 8).

## 5. Conclusions

**Figure 18:**
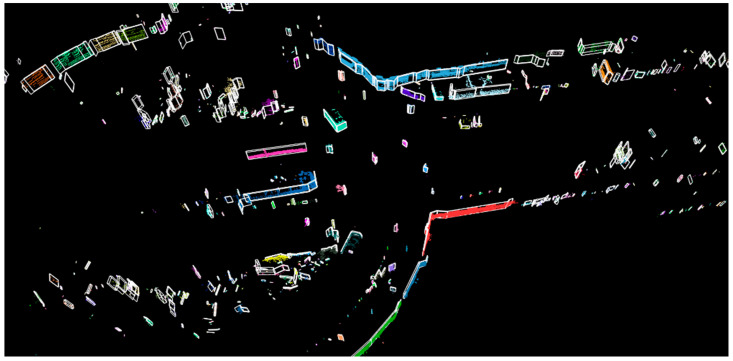
Facet representation on a complex scene.

In conclusion, a framework for obstacle facet detection from LiDAR data was presented (final results in Figure 18). As contributions, the proposed framework improves existing approaches (as for ground detection, where the speed is increased) or proposes new methods for obstacle clustering and obstacle facet detection. For obstacle clustering, we proposed an efficient (runtime and memory) method based on direct grouping of points inside each channel and between neighboring channels. The proposed algorithm for facet detection scans the obstacle contour and extracts relevant facets. All methods were evaluated using existing benchmarks. The quantitative results demonstrate the advantages of the proposed improvements.

The quality of the results is dependent on the performance of the ground point detection step, but for this step, more robust methods can be used (especially for complex road geometries). Another difficult scenario is when several objects are very close to each other, and a single cluster might be detected. For this, a potential solution is to use additional camera data and semantic or instance segmentation. However, it is important to mention that, even if several objects are clustered together, the facet representation provides an accurate occupied area that follows the contour of the cluster.

For facet detection, further development ideas are determining the key points of the contour in a sliding window to have fewer points to process, determining the main angle of orientation for each object, and also evaluation improvement (including the creation of better clusters). A learning-based approach might be developed in the future for facet extraction, but the main challenge is the lack of ground truth data (and the difficulty to build such a data set).

## Figures and Tables

**Table 1:** Values of the parameters used in the proposed framework.

Parameter	Value
Number of channels	1800
DISTANCE_BETWEEN_CLUSTERS	0.15 m
PREVIOUS_CHANNELS_TO_CHECK	7
Maximum valid intra-clusters	50
MAX_DISTANCE_TO_FACET	0.08 m
MAX_CONSECUTIVE_OUTLIERS	4
ANGLE_DIFF	10°
Maximum number of facets for an object	100
Maximum RANSAC iterations	20

**Table 2:** Ground detection: values for each type of value using the evaluation metrics (based on 252 scenes, entire 360° point cloud).

Type	Experimental Results of [3]	$\mathrm{With}\;tan^{-1}$
True positive (TP)	17267627	17268115
True negative (TN)	11586608	11586615
False positive (FP)	730193	729710
False negative (FN)	755548	755536

**Table 3:** Ground detection: values for each evaluation metric (using data from Table 2).

Metric	Experimental Results of [3] (%)	$\mathrm{With}\;tan^{-1}(\%)$
Accuracy	95.10	95.10
Precision	95.94	95.94
Recall	95.80	95.80
f1-score	95.87	95.87

**Table 4:** Ground detection: runtime comparison (based on 252 scenes, entire 360° point cloud).

	Method	Serial (ms)	Parallel—4 Threads (ms)
Minimum	$\sin^{-1}$	5.77	2.01
	$\tan^{-1}$	4.47	1.90
Average	$\sin^{-1}$	7.34	2.93
	$\tan^{-1}$	**6.10**	**2.78**
Maximum	$\sin^{-1}$	8.35	3.76
	$\tan^{-1}$	7.30	3.47

**Table 5:** Clustering: runtime comparison (based on 252 scenes, entire 360° point cloud).

	Octree (ms)	Octree Parallel (ms)	Proposed Method (ms)	Proposed Method Parallel (ms)
Minimum	20.00	9.48	8.00	5.08
Average	42.02	29.06	**11.50**	**6.72**
Maximum	167.03	79.27	18.95	8.73

**Table 6:** IoU scores obtained on the tested methods (based on 7481 scenes from KITTI object detection).

Type	Proposed Solution (%)	Adapted [34] (%)
Car	**71.27**	70.56
Cyclist	**51.25**	49.92
Misc	**57.18**	57.17
Pedestrian	38.24	**38.25**
Truck	72.35	**72.36**
Van	**71.66**	70.99

**Table 7:** Serial runtime for facet detection: adapted [34] vs. proposed method (based on 252 scenes, entire 360° point cloud).

	Adapted [34] (ms)	Proposed Method (ms)
Minimum	32.00	**5.75**
Average	53.72	**10.83**
Maximum	132.98	**25.58**

**Table 8:** Runtime for proposed solution (facet detection) on parallel execution (based on 252 scenes, entire 360° point cloud).

	Proposed Solution-Parallel Execution (ms)
Minimum	1.92
Average	3.83
Maximum	8.49
